# Extension of the yeast metabolic model to include iron metabolism and its use to estimate global levels of iron‐recruiting enzyme abundance from cofactor requirements

**DOI:** 10.1002/bit.26905

**Published:** 2019-01-12

**Authors:** Duygu Dikicioglu, Stephen G. Oliver

**Affiliations:** ^1^ Department of Chemical Engineering and Biotechnology University of Cambridge Cambridge UK; ^2^ Cambridge Systems Biology Centre, University of Cambridge Cambridge UK; ^3^ Department of Biochemistry University of Cambridge Cambridge UK

**Keywords:** enzyme cofactor turnover, iron metabolism, iron‐sulphur maturation, metabolic networks, yeast

## Abstract

Metabolic networks adapt to changes in their environment by modulating the activity of their enzymes and transporters; often by changing their abundance. Understanding such quantitative changes can shed light onto how metabolic adaptation works, or how it can fail and lead to a metabolically dysfunctional state. We propose a strategy to quantify metabolic protein requirements for cofactor‐utilising enzymes and transporters through constraint‐based modelling. The first eukaryotic genome‐scale metabolic model to comprehensively represent iron metabolism was constructed, extending the most recent community model of the *Saccharomyces cerevisiae* metabolic network. Partial functional impairment of the genes involved in the maturation of iron‐sulphur (Fe‐S) proteins was investigated employing the model and the in silico analysis revealed extensive rewiring of the fluxes in response to this functional impairment, despite its marginal phenotypic effect. The optimal turnover rate of enzymes bearing ion cofactors can be determined via this novel approach; yeast metabolism, at steady state, was determined to employ a constant turnover of its iron‐recruiting enzyme at a rate of 3.02 × 10 ^−11^ mmol·(g biomass) ^−1^·h ^−1^.

## 1 INTRODUCTION

Metabolic networks are comprised of the interactions of metabolites, enzymes and their regulators (Sauer, 2006). Just two of these components, the enzymes and the metabolites, are included in most metabolic network models. Enzyme abundance and metabolite concentrations have been shown to act inversely to maintain homeostatic control of metabolic reaction rates in *Saccharomyces cerevisiae* (Fendt et al., 2010). Thus an understanding of their relationship can help us to determine the nature of transitions between different metabolic states.

The measurement of intracellular metabolite pools and protein abundances are useful approaches to understand these homeostatic mechanisms, and have been applied to many different systems ranging from bacteria such as *Escherichia coli* (Bennett et al., 2009) to tumour cell lines (Madhukar, Warmoes, & Locasale, 2015; Matsumoto et al., 2016). Although total protein content and absolute quantification of individual proteins have been reported for a number of biological systems (Bennett et al., 2009; Carroll et al., 2011; Madhukar et al., 2015; Matsumoto et al., 2016; Picotti, Bodenmiller, Mueller, Domon, & Aebersold, 2009), the properties of metabolic systems cannot be defined by studying enzyme proteins in isolation. Enzymes are often present in lower copy numbers compared to other components of the proteome, such as ribosomal proteins. For some enzymes, these copy numbers are at the limits of detection and quantification by current analytical techniques (Picotti et al., 2009). A recent study on the direct and absolute quantification of over 1,800 yeast proteins revealed that <25% of the proteins that could be quantified were components of the metabolic network (Lawless et al., 2016).

Metabolic models with high predictive ability are important tools for the investigation and engineering of metabolism (Aung, Henry, & Walker, 2013). Metabolic models can provide reasonable predictions when direct measurements of network components are infeasible, as is demonstrated by predictions on flux distributions using genome‐scale models (Orth, Thiele, & Palsson, 2010). Analyses with stoichiometric models can be used to predict flux, but only a limited number of studies exist on their use for estimating absolute enzyme abundances, for example in yeast (Nilsson & Nielsen, 2016; Sánchez et al., 2017); an alternative approach would be to make use of the cofactors to determine the relative abundances of those enzymes that use those cofactors.

To exploit this relationship between metabolic enzymes and their cofactors, we propose a network‐based strategy to determine optimal enzyme abundance by model predictions based on the metabolic requirements of their cognate cofactors. We have studied a family of cofactors that are not, themselves, metabolic intermediates and which do not have donor functional groups. We chose to study the iron cofactor family, including iron in its ionic form (Fe^3+^, Fe^2+^) and complex ion entities such as the haem family (sirohaem, haems A, B, C and O), and the iron‐sulphur clusters (2Fe‐2S, 4Fe‐4S), because these iron‐containing cofactors bind nearly 10% of the documented enzymes and transporters in the metabolic network. Baker’s yeast, the first eukaryote to have its genome sequenced (Goffeau et al., 1996), has long been a favourite model organism (Botstein, Chervitz, & Cherry, 1997). Cellular activities including DNA replication, recombination and repair, RNA transcription and translation, intracellular trafficking, as well as the enzymatic activities of general metabolism, and mitochondrial biogenesis are conserved from yeast to human (Barrientos, 2003). The availability of comprehensive and powerful genome‐scale models of the yeast metabolic network (Aung et al., 2013) for almost two decades (Famili, Forster, Nielsen, & Palsson, 2003) made yeast an ideal model for our study. Although there is a wealth of knowledge about iron utilisation and homoeostasis in yeast (De Freitas et al., 2003; Lill & Mühlenhoff, 2008; Miethke & Marahiel, 2007), this information has not been integrated into the curated genome‐scale metabolic model, thus limiting the model’s usefulness for in silico studies. This issue has recently been highlighted by a comprehensive study on the comparative analysis of yeast metabolic models (Heavner & Price, 2015).

In this study, we have extended the genome‐scale metabolic model of *Saccharomyces cerevisiae* to include iron metabolism, and made the first comprehensive mathematical representation of any inorganic ion in a model of a network in a eukaryote; this model is publicly available (BioModels [Chelliah et al., 2015] access no: MODEL1709260000). The new model has been benchmarked against known environmental responses (viz., changes in the availability of iron and copper) and genetic modifications relating to iron metabolism. The model has allowed the identification of the extensive rewiring of metabolic fluxes to cope with the hemizygosity of essential genes involved Fe‐S cluster maturation. The model permitted the establishment of the requirements for iron‐family cofactors, based on how the fluxes were distributed through the metabolic network. These requirements were then used as proxies for calculating the metabolic requirement of those metabolic enzymes and transporters that employ iron species as cofactors.

## 2 MATERIALS AND METHODS

### 2.1 Modelling methods

#### 2.1.1 Primary metabolic model, simulation environment and model annotation

The primary model for the incorporation of iron metabolism was selected as the most recent stoichiometric model of the *S. cerevisiae* metabolic network (v7.6; Aung et al., 2013). The extended model (Yeast7.Fe) is provided as Supporting Information S1 and the details on modification and extension of the existing model are provided in Supporting Information S2 in supplemental material. The extended genome‐scale model of *S. cerevisiae* is available in a COBRA compatible‐SBML format (v.4). A specific growth rate of 0.1 hr^−1^ was used unless otherwise specified. The iron and copper supplementations of the system was determined from a standard defined medium for *S. cerevisiae* (Baganz, Hayes, Marren, Gardner, & Oliver, 1997). Iron and copper limitations were imposed on the system by scaling the extracellular availability of the respective ions in the medium down to 10% of the original upper bound. Details regarding the extension annotations and the simulation environment and approaches are provided in Supporting Information S3, and the simulation code is provided in Supporting Information S4.

#### 2.1.2 Cofactor representation

For any metabolic reaction catalysed by a cofactor requiring enzyme (*N*):

aA+bB⇋cC+dD,

where the stoichiometric coefficients of the substrates (*A* and *B*) and products (*C* and *D*) are denoted by their cognate lower case letters, a cofactor (*X*) of enzyme *N* enters the metabolic reaction as a substrate and leaves as a catalytically unreactive product:

aA+bB+xX⇋cC+dD+xX.

Details regarding the representation of cofactors are provided in Supporting Information S3. The distribution of fluxes throughout the metabolic network would be correctly influenced by the requirements for, and the availability of, iron in this representation. Iron‐containing cofactors could be required for some of the enzymes necessary for catalysing a reaction, and a dedicated amount of cofactors should be reserved for this use, without being actually involved in the reaction itself. The effect that this modification had on the distribution of fluxes, including the growth rate, will be discussed in Section 3.

#### 2.1.3 Evaluation of the predictive power of the model

Prediction of gene essentiality was used as the evaluation criterion for the new reconstruction. Empirical data on *S. cerevisiae* S288C strain essentiality was obtained from Saccharomyces Genome Database (SGD; Cherry et al., 2012; website accessed on March 22, 2017). A full list of non‐SGD resources for gene essentiality is provided in Supporting Information S5 in supplemental material. Details regarding evaluation of the predictive power of the model are provided in Supporting Information S3.

### 2.2 Experimental methods

#### 2.2.1 Strains, cultivation conditions, subcellular fraction enrichment protocols and analytical assays

Heterozygous deletion mutants *HO/Δho*, *ARH1/Δarh1*, *ATM1/Δatm1* and *YFH1/Δyfh1* of *S. cerevisiae* strain BY4743 (background: *MAT**a**/Δ his3Δ1/his3Δ1 leu2Δ0/leu2Δ0 lys2Δ0/LYS2 MET15/met15Δ0 ura3Δ0/ura3Δ0* ) were used in this study (Baker Brachmann et al., 1998). Deletion of a single copy was verified by PCR using the confirmation primers described in Baker Brachmann et al. (1998). Qiagen DNeasy Blood & Tissue Kit was used for isolation and purification of DNA from the cell extracts as described in the manufacturer’s protocol. Analytical assays were carried out employing enzymatic or colorimetric methods. Details regarding those analytical assays, as well as those of the cultivation conditions and the subcellular fraction enrichment protocols are provided in Supporting Information S3. All data pertaining these analyses are provided in Supporting Information S5 in supplemental material.

## 3 RESULTS

### 3.1 Iron metabolism in yeast metabolic models

**Figure 1:**
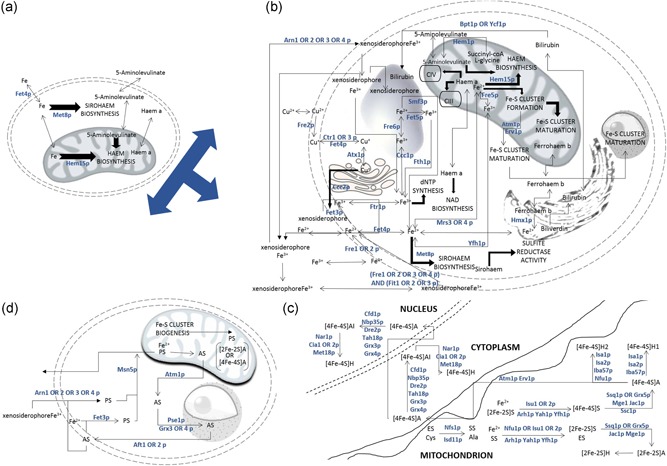
Schematic representation of iron metabolism in the yeast model. Pathways and metabolites are represented by uppercase and lowercase letters, respectively. Directionality of the fluxes through the pathway is specified by arrows (for single reaction steps) and block arrows (for lumped consecutive reaction steps). Metabolic enzymes are shown in teal colour. The cell and organelle boundaries are represented as double dashed lines; the mitochondrion, nucleus, vacuole and ER have cartoon representations. The minimal representation of iron metabolism in the existing Yeast 7.6 model is provided in (a). The details on the reductive, non‐reductive and xenosiderophore‐bound iron uptake, intracellular transport and storage of iron, haem and sirohaem biosynthetic and degradation pathways are provided in (b). Details regarding the biogenesis of Fe‐S clusters in the mitochondrion (ISC machinery), and the maturation of apoenzymes (A) into Fe‐S cluster‐bound holoenzymes (H) in the mitochondrion (ISC machinery), in the cytosol and in the nucleus (CIA machinery) are provided in (c). An empty scaffold (ES) and its sulphonylated form (SS) were introduced as pseudometabolites in the Fe‐S cluster formation mechanism. The regulation of iron uptake via the iron regulon, employing the negative feedback from Fe‐S cluster biogenesis, is demonstrated in (d). The shuttling of the signals representing the availability of mitochondrial iron (PS) and its depletion (AS) were introduced as pseudo‐metabolites to modulate and activate the reductive iron uptake routes. For simplifications of the function and activity of the iron regulon, see text [Color figure can be viewed at wileyonlinelibrary.com]

Although the predictive power of the yeast metabolic network model has improved substantially over the years, it is still limited by the omission or incomplete representation of some pathways in the model. Our previous work on the Yeast 7 metabolic network model highlighted the fact that the high metabolic burden (characterised by high reaction fluxes, and often indicative of the low efficiency of their cognate enzymes (Bonarius, Hatzimanikatis, Meesters, Schmid, & Tramper, 1996) carried by pathways involved in energy generation and processes imposed limitations on the model’s predictive ability (Dikicioglu, Kırdar, & Oliver, 2015). A recent analysis that we carried out by constraining this model by fluxes calculated using the intracellular concentrations of intermediates in the purine nucleotide biosynthetic pathway, as determined by HPLC analysis (Hesketh, Vergnano, Wan, & Oliver, 2017), demonstrated that the prediction of growth rate was at least half or twice the experimentally determined value. The distribution of the fluxes indicated that the iron uptake and utilisation pathways were inactive because they were inadequately represented in the model and completely disconnected from the rest of the metabolic network (Figure 1a). We addressed each of the deficiencies through extensive literature curation and will explain how iron metabolism was incorporated into the network model in the following sections.

#### 3.1.1 Uptake, intracellular transport and storage of iron

Both reductive (Fe^3+^—transporting, high affinity) and non‐reductive (Fe^2+^—transporting, low affinity) iron uptake mechanisms were incorporated into the metabolic network. High‐affinity iron uptake, as both free and xenosiderophore‐bound iron, was represented by the reductive pathway. The intracellular transport of iron and complex iron entities, such as haem and sirohaem, across intracellular boundaries and the storage of residual components and excess iron were also considered (Figure 1b). Iron export is not known to be exhibited by *S. cerevisiae* (Haas, Eisendle, & Turgeon, 2008) and so was excluded.

The concentration limit to determine the lower bound of the flux through the low‐affinity iron uptake reactions was set at 1 µM (Lesuisse, Blaiseau, Dancis, & Camadro, 2001). The high‐ and low‐affinity uptake systems were modelled as working independently of one another. However, in the absence of a functional low‐affinity system, the high‐affinity system will be used, even in the presence of abundant iron (and vice versa); these situations are allowed for in the model (Haas et al., 2008; Lesuisse et al., 2001). Specific ARN family transporters involved in the uptake of each iron‐bound xenosiderophore and the fate of each xenosiderophore in the yeast cell was modelled individually for ferrichrome, *N*,*N′*,*N″*‐triacetylfusarinine C (TAFC), enterobactin and ferrioxamine B (see Supporting Information S2 in supplemental material; Haas et al., 2008). The reductive assimilation of iron bound to xenosiderophores was facilitated by one of the functionally non‐interchangeable metalloreductases (Fre1p–4p; Yun, Bauler, Moore, Klebba, & Philpott, 2001) and all members of the family of mannoproteins (Fit1p–3p) that are incorporated into the cell wall via glycosylphosphatidylinositol (GPI) anchors in *S. cerevisiae* (Haas et al., 2008).

The mechanism of copper recruitment by the reductive pathway of high‐affinity iron uptake (Philpott, 2006) necessitated the incorporation of copper uptake into the metabolic network. Since copper metabolism was not represented in the primary metabolic network of yeast to any degree, the network was extended to incorporate the uptake of copper and its function in high‐affinity iron uptake. It is important to note that the representation of copper was not exhaustive; only activities that are relevant to iron metabolism were considered in this reconstruction. The threshold for switching between high‐ and low‐affinity copper transport was set as 20 µM (Hassett, Dix, Eide, & Kosman, 2000). The threshold concentrations were used to calculate a threshold flux boundary to be used in the model. Two assumptions were made in modelling the uptake of copper across the cell envelope: (a) Although included as a unique species in the model, the high‐affinity copper transporter Ctr3p was not associated with the copper uptake reaction in the model along with Ctr1p. This exclusion was necessary since the *CTR3* gene, found in strains of the S288C lineage that were used in this study, has been inactivated by insertion of a Ty2 transposon (Knight, Labbé, Kwon, Kosman, & Thiele, 1996). (b) Fre1p copper reductase was similarly excluded from gene‐reaction associations since it was reported to be active only during the first 3–4 hr post‐inoculation (Georgatsou & Alexandraki, 1994). The in silico analyses conducted in this study were all carried out using pseudo‐steady‐state assumption; by that time, Fre2p was thought to determine copper reductase activity.

#### 3.1.2 Biosynthesis and recycling of complex iron entities

The biosynthesis and recycling of haem, sirohaem and Fe/S clusters were fully implemented in the genome‐scale model of the yeast metabolic network. The synthesis of 5‐aminolevulinate from glycine and succinyl‐CoA via the Shemin pathway (Ferreira & Gong, 1995) was introduced and 5‐aminolevulinate uptake was excluded from the model. Haem catabolism in the endoplasmic reticulum, and the storage of bilirubin in the vacuole were also introduced to the model de novo (Figure 1b).

A pseudometabolite—mitochondrial empty scaffold (ES)—was introduced to the metabolic network to model Fe‐S cluster biogenesis (the ISC machinery) in the mitochondria and Fe‐S cluster assembly (the CIA machinery) in the cytosol and nucleus. This allowed the maturation of the Fe‐S clusters and the ensuing transfer to their cognate apoenzymes (Johnson, Dean, Smith, & Johnson, 2005; Lill & Mühlenhoff, 2006; Lill et al., 2012; Urzica, Pierik, Mühlenhoff, & Lill, 2009). Although this scaffold is thought to be a transient protein complex (Lill & Mühlenhoff, 2008), we associate the members of this complex with a “reaction” step in the metabolic model that converts an empty scaffold into a sulphonylated scaffold (SS). The ES is then released in the next step once the Fe‐S cluster itself was formed. Both 2Fe‐2S and 4Fe‐4S cluster maturation have been assigned to the mitochondrion, cytosol, or nucleus as applicable. Two different mitochondrial 4Fe‐4S maturation routes have been implemented to account for the genetic determination of 4Fe‐4S cluster maturation for lipoic acid synthesis (by Lip5p) and succinate dehydrogenase (encoded by *SDH2*) or for other mitochondrial Fe‐S proteins (denoted as H1 or H2 in Figure 1c, respectively; Lill & Mühlenhoff, 2008).

#### 3.1.3 The iron regulon

Two different regulatory mechanisms for iron uptake via the iron regulon were implemented in this model: (a) the yeast‐specific haem‐regulated positive feedback route (Lill & Mühlenhoff, 2008), and (b) the mitochondrial Fe‐S cluster biogenesis‐associated negative feedback route.

Haem deficiency, indicated by the deactivation of the enzymes encoded by the essential genes (*HEM1–4*, *HEM12*, *HEM13*, *HEM15*) of the pathway, was reported to be an indicator of low‐iron uptake in yeast (Lill & Mühlenhoff, 2008). We coupled the essential enzymes of haem biosynthesis to the reaction representing low‐affinity iron uptake catalysed by Fet4p to account for this positive feedback mechanism by associating these essential enzymes with the low‐affinity iron uptake reaction. This coupling with low‐affinity iron uptake ensured that haem biosynthesis would not be over‐activated by the network unless extracellular iron was abundant.

The negative feedback on iron uptake by Fe/S cluster biogenesis is shut down upon iron depletion. This was modelled by introducing two new pseudo‐metabolites: AS signalling the depletion of intracellular iron, and PS indicating the intracellular availability of iron (SBO:0000409 term: interaction outcome). AS activated the iron regulon in the nucleus, relaying a message to the cell envelope to activate the uptake of iron (Figure 1d). PS was coupled with the formation of Fe‐S apoclusters in the mitochondrion. This was done by introducing PS as a metabolite in the Fe‐S cluster biogenesis reaction, thus ensuring that the reaction would have flux as long as PS, that is iron, was available. Unavailability of iron produced AS, which was then relayed from the mitochondrion to the nucleus and further to the cell envelope, unchanged. Msn5p, a karyopherin shuttling between the nucleus and the cytoplasm, was assigned a modified function in the model, being associated with the transport of PS from the cell envelope back to the mitochondrion, thus representing the relay of the signal for the presence of iron in the cell. Aft1p, which is responsible for relaying the iron depletion signal to the cell boundary, was reported to have an additional role in creating iron resources for the cell by binding Cth2p to facilitate the degradation of the mRNAs for iron‐containing enzymes (Cherry et al., 2012). This route was excluded from the network since the model does not treat enzymes as either the substrates or products of reactions. Aft1p was also reported to mediate saving iron by activating Vth1p‐mediated biotin uptake since biotin synthesis was reported to be iron consuming (Shakoury‐Elizeh et al., 2004). This route was also excluded since biotin co‐enzyme metabolism was not considered to be of direct interest to our system.

### 3.2 Iron family cofactor considerations of metabolic enzymes

Iron family cofactors, copper ions and pyridoxine (the last is used in only one reaction) were used as substrates and untransformed reactants in those reactions that were catalysed by enzymes activated by these cofactors, as described in “*Cofactor representation*.” This allowed us to establish the connectivity between iron metabolism, described above, and the primary metabolic network of yeast. For this purpose, the copper, iron, haem, sirohaem, pyridoxine and Fe‐S requirements of the metabolic network were identified (see Supporting Information S6 and S7 in supplemental material; De Freitas et al., 2003; Johnson et al., 2005; Lill et al., 2012; Terali, 2010). A variety of reasons led us to exclude 41 enzymes reported to involve iron cofactors from this compilation: the documented information was observed to conflict with other available information; the enzyme was not included in the Y7.6 version of the yeast metabolic network model, or the protein was associated with a regulatory task (see Supporting Information S8 in supplemental material; De Freitas et al., 2003; Johnson et al., 2005; Lill et al., 2012). The metabolic enzymes with iron‐binding attributes in the reconstruction Y7.Fe included all metabolic enzymes whose empirically verified non‐ubiquitous iron‐binding properties were reported in UniProt, and which were assigned to the iron‐ion binding Molecular Function Gene Ontology (GO:0005506; both databases accessed on August 1, 2018). The reconstruction captured 24 (60% of existing empirical data), including such enzymes identified through literature mining and curation additional to those reported in the public databases (see Supporting Information S9 in supplemental material for detailed information on these comparisons).

### 3.3 Consideration of cell growth by indirect association with the biomass equation

Although the elemental iron content of yeast has long been determined (Lange & Heijnen, 2001), experimental data are not available on how much iron content existing yeast biomass constituents possess or on the relative content of potential key iron‐containing compounds that could potentially be included, and therefore, it was not possible to incorporate any direct involvement of iron into the biomass equation. Given this dearth of available data, simply adding an iron ion term would result in the iron flux being syphoned off towards biomass, bypassing the metabolic network. Even without a representation of the iron content in biomass components, our model is able to predict the essentiality of iron for the cell indirectly, and correctly predicts that yeast becomes inviable upon complete depletion of iron from the extracellular environment.

Given the above limitations, and following the conduct of a *Gedankenexperiment*, we propose an approach for the incorporation of iron species in the definition of biomass, and consequently, in the biomass equation of the metabolic network. For this purpose, we identified which amino acids bind different types of iron entities and in what ratios (Table 1). The relative abundances of proteins that have functional iron entities in comparison to the global proteome were extracted from Paulo et al. (2016). The stoichiometric coefficients of the iron entities to be incorporated into the biomass equation were calculated from the stoichiometric coefficients of the amino acids that could potentially bind (see Supporting Information S10 for details on the calculation).

**Table 1:** Amino acid—iron entity binding relationships in biomass definition

Iron entity	Attached amino acid	Binding ratio per iron entity	Reference
4Fe‐4S	Cysteine	2	Andreini, Bertini, Cavallaro, Najmanovich, and Thornton (2009)
4Fe‐4S (biotin synthase)	Arginine	2	Andreini et al. (2009)
2Fe‐2S (Rieske)	Histidine–cysteine	2–2	Andreini et al. (2009)
2Fe‐2S (non‐Rieske)	Cysteine	4	Andreini et al. (2009)
Haem b	Cysteine	1	Li, Bonkovsky, and Guo (2011)
Haem c	Cysteine	2	Li et al. (2011)
Fe(III)‐mono	Cysteine	4	Andreini et al. (2009)

### 3.4 Reconstruction of the extended stoichiometric model and general design considerations

Reconstruction of the model involved the modification of four existing species (all metabolites), 13 species types (11 metabolites and two enzymes), and 53 existing reactions, as well as the removal of three reactions from Y7.6. Some 90 species types (68 enzymes, 18 metabolites and four pseudo‐metabolites), representing 173 species (67 enzymes, 11 pseudo‐metabolites and 95 metabolites) and 104 new enzymatic and transport reactions were introduced to the model. Here, “species” denotes both metabolites and enzymes as the standard nomenclature adopted in the model. Thus the new version of the model, Y7.Fe, has improved the gene coverage of the existing model by 6%.

Reactions, which were reported to take place at the membrane but did not have a detailed mechanism explained, were represented to occur across the membrane. This device simplified the model without compromising on the details of iron metabolism. The enzymes associated with these reactions were localised to the membrane to highlight the transmembrane nature of the reaction. Pseudo‐metabolites were introduced such that they did not interfere with the material, energy, or redox balances of the network. They were introduced in coupled reactions to avoid accumulation, and these reactions were unbounded so that the system was not constrained by the flux limitations through these reactions when the underdetermined system was optimised for a given objective.

Only 14 new dead‐end metabolites could be detected in Y7.Fe, indicating that the extension by incorporation of iron metabolism did not disrupt the connectivity of the metabolic network. All dead‐end metabolites were new species in the iron model, and were individually curated for their use and functionality within the network. The Fe‐S clusters matured in the nucleus are not recruited by the metabolic enzymes in the current reconstruction. However, this process was included to enable future extensions of the model, albeit at the cost of introducing phosphate and mature 4Fe‐4S species as dead‐end metabolites in the nucleus. Other dead‐end metabolites were introduced through cyclic interconversions: PS (extracellular), NADPH, NADP, FMN and FMNH_2_ (in vacuole) and FMNH_2_ (in the mitochondria). Some by‐products of iron metabolism are allowed to accumulate in the yeast cell as reported in the literature: bilirubin and ferrioxamineB (in the vacuole), coprogen, ferrichrome and enterobactin (in the cytoplasm). l‐Cysteine is transported into the mitochondrion in our model for the assembly of the sulphonylated scaffold for Fe‐S cluster formation. Although recent reports have suggested a putative cysteine synthase (Mcy1p) identified in the mitochondrial outer membrane (Hughes, Hughes, Henderson, Yazvenko, & Gottschling, 2016), this mechanism has not yet been extensively investigated and so was excluded. The biomass‐modified version of the model (Y7.FeBM; Supporting Information S11) had the same number of dead ends and yielded similar predictions on growth rate to the original model reconstructed in this study.

Some 97% of the reactions contained in Y7.Fe were comparable to those of Y7.6 and only 823 (ca. 23%) reactions had non‐zero fluxes when the unit glucose uptake rate was used as the single constraint for optimising growth. Of those 823 reactions, the flux through only 229 remained the same and the difference in fluxes through 254 of the remaining reactions was more than 25%. This indicated that introduction of iron metabolism into the genome‐scale metabolic model resulted in a substantial rewiring of nearly one‐third of the active reactions in the model under standard growth conditions. The predicted growth flux was reduced by 7% in Y7.Fe (see Supporting Information S5 in supplemental material), indicating the cost of the iron metabolism to the existing network. This high metabolic burden was most likely to be associated with energy generation, and could not be captured by Y7.6. This observation was in line with the growth predictions obtained by constraining the fluxes through the purine pathways in our preliminary analysis. Revisiting the same system, the flux predictions improved substantially employing Y7.Fe with only a ±20% difference between the experimentally measured and predicted growth fluxes, at the cost of lower predictive accuracy of the growth phenotype. Indeed, an analysis of those reactions, which displayed more than 25% change in their flux between Y7.6 and Y7.Fe, showed that they were associated with genes that were significantly enriched for the nucleoside phosphate metabolism process term (*p* < 10^–38^) along with other metabolic processes in line with our observations on the analysis involving the purine intermediates used as flux constraints. We carried out a sensitivity analysis to investigate robustness by selecting the fluxes that displayed a change more than 15–35% (in 5% increments), and observed that the genes encoding the enzymes that catalysed these reactions were significantly enriched for the same, or very similar processes (see Supporting Information S5 in supplemental material). The variability of the flux distributions was taken into consideration in conducting this analysis (for details of the method, see Supporting Information S3 in supplemental material).

The analysis of the fluxes in the biomass‐modified model, Y7.FeBM, indicated that the magnitude of only 8% of the fluxes were altered as a response to this change and that the magnitude of the change was minimal (Supporting Information S10). The modifications were proportional enrichment in the fluxes for the production of the iron entities that were incorporated into the biomass equation. Despite being far from providing a complete picture due to the problems regarding data availability discussed above, this exercise of incorporating iron entities into biomass definition demonstrated that, in fact, it was the incorporation of the iron metabolism into the network that caused extensive rewiring of the fluxes when iron was taken into consideration as a metabolic cofactor, rather than the part Fe‐proteins played in the biomass.

The predictive power of the Y7.Fe model was further investigated by determining its ability to define gene essentiality. The extended model was observed to perform very similarly to the existing primary model, Y7.6, with only marginal differences in measures evaluating predictive power of the model despite a sizeable improvement of 6% in gene coverage (Table 2). Major improvements and extensions in the metabolic network model structures were previously observed to have substantial negative effects on the predictive power as a trade‐off (Aung et al., 2013). Both Y7.Fe and Y7.6 models performed similarly in predicting gene essentiality, indicating that the additional 14 dead‐end metabolites introduced in our reconstruction did not affect the quality of the existing model.

**Table 2:** Evaluation of the predictive power of the extended model

	Y7.6	Y7.Fe
Number of genes	908	963
Number of TP	677	709
Number of TN	83	84
Number of FP	73	90
Number of FN	76	80
PPV (%)	90	89
NPV (%)	52	48
Sensitivity (%)	90	90
Specificity (%)	53	48
Predictive success (%)	84	82

### 3.5 Iron‐recruiting enzyme requirements of the metabolic network model

**Figure 2:**
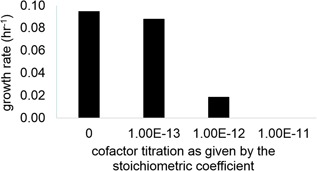
Indispensable role of iron for yeast. This plot demonstrates how growth rate predictions of the metabolic network model are affected by the amount of iron recruited by the metabolic enzymes. The stoichiometric coefficient of iron ions and complex iron entities to be recruited by iron requiring enzymes without impairing growth substantially can be determined based on the constraints imposed by the experimentally permissible limits of iron uptake

The stoichiometry of the cofactors introduced into the reactions was observed to be closely related to the predictions of growth rate, which meant that such predictions were very sensitive to the metabolic requirement for iron in the network. The total iron requirement of the cell was calculated from the iron composition of a standard defined medium for yeast (Chiu & Segrè, 2008), and the recruitment of iron or complex iron entities as cofactors by the enzymes was simulated based on the constraint imposed by the iron transport flux. Fine‐tuning for the sustainable iron recruitment indicated that the stoichiometric coefficients for these terms need to be in the order of magnitude of 10^–14^ if the supply of iron is not to reduce the rate of growth (Figure 2). The metabolic requirement of total turnover for iron‐recruiting enzymes was determined as 3.02 × 10^–11^ mmol·(g biomass)^−1^·h^−1^ based on this model. The maximum and minimum theoretical requirements for iron‐recruiting enzymes were determined by investigating the variability of these fluxes. This analysis demonstrated that the cell employs the pathways where minimal iron recruitment of the cognate enzymes would be required, possibly to improve the energy efficiency of the system. The total turnover was only marginally (0.02‰) higher than that computed for the theoretical minimum usage. On the other hand, we observed that the fluxes through these pathways would be rewired such that the turnover of iron‐recruiting enzymes would be increased by 34% (Supporting Information S5).

The stoichiometric coefficients for cofactors were determined to be very low, as expected by their biological context. Although iron was not among the trace element cofactors, the stoichiometric coefficients were determined to be in the magnitude of 10^–14^ (see Section 2.1.3). An alternative strategy to determine enzyme abundances from the reaction flux and the turnover rate of these enzymes (*k*_cat_) could be evaluated to replace the stoichiometric coefficient strategy. Iron, being a micronutrient, is usually provided in the growth medium at a low concentration. Therefore, constraining enzyme abundances by iron availability constrains the reaction flux altogether, since the *k*_cat_ for each reaction would remain constant. Even making a favourable selection of *k*_cat_ values based on available data, the constraint on iron availability indicated that material flow through high‐flux reactions, such as those involved with the mitochondrial respiratory chain, would then adopt impractically low values to satisfy the constraint on iron availability and compromise growth rate substantially. This indicated that the limiting factor concerning iron metabolism was the availability of iron, not the enzyme turnover rates, necessitating the modulation of reaction stoichiometry.

Working with such small numbers also imposes technical constraints on the analysis. The precision of the machine and the solver used can both cause problems at this stage. For the current analyses, the precision of both the machine and Gurobi (Gurobi Optimizer Version 3.0; Gurobi Optimization, Inc., Houston, TX; April 2010, http://www.gurobi.com/) were two orders of magnitude lower than the stoichiometric coefficients, allowing us a robust platform to conduct the analyses independent of whether the MATLAB or the Python version of Cobra was utilised. Several tests were run to ensure that Gurobi did not treat the stoichiometric coefficients as zero, and that the performance of the solver was not compromised (as described in Supporting Information 3). However, for less abundant cofactors, this is an issue that needs to be addressed.

### 3.6 Benchmarking the model against environmental and genetic challenges

Having established the iron‐recruiting enzyme requirement of the metabolic network, we extended the investigation to understand how the in silico system adapted to environmental challenges that were tailored specifically to exploit this model. The response of the network to different levels of extracellular iron and copper availability was investigated through flux distributions. For this purpose, the upper and lower bounds of the fluxes through several reactions were set to zero to mimic reports available in the literature (Haas et al., 2008; Lesuisse et al., 2001). The high‐affinity iron uptake system was activated by blocking the routes through low affinity Fe^2+^ uptake routes. The absence of extracellular xenosiderophores was also taken into consideration for the simulation of an *S. cerevisiae* monoculture. The predictions on flux distributions were in agreement with the expected physiological outcome on how the yeast metabolism responded to low or high abundance of copper (Cankorur‐Cetinkaya et al., 2013; Vest et al., 2016) or iron (Holmes‐Hampton, Jhurry, McCormick, & Lindahl, 2013; Vest et al., 2016; Table 3).

**Table 3:** Performance table for benchmarking the model predictions with empirical observations^a^

	Y7.Fe predictions		Empirical observations	
	O_2_ uptake	Growth	O_2_ uptake	Growth
High iron available in the extracellular space	↔	↔	↔	↔
Low iron available in the extracellular space	↓	↓	↓	↓
High copper available in the extracellular space	↔	↔	↔	↔
Low copper available in the extracellular space	↔	↔	↔	↔
Hemizygosity in *ARH1*	↓	↔	↓	↔
*Δccc2/ Δccc2*	↓	↔	↓	↔
*Δccc2/ Δccc2*—no copper supplementation	↔	↓	↔	↓

^a^ ↑: increase against control; ↓: decrease against control, ↔ : remains constant.

We then investigated how Y7.Fe could be used to study the impact of the deletion of the genes *ARH1*, *ATM1* or *YFH1* or a reduction in the functionality of their protein products, which are all essential components of the ISC machinery. All three genes are essential, although only *arh1* deletants could be identified as inviable employing the model. This implies that *YFH1* (the Fe‐S cluster scaffold protein) and *ATM1* (the Fe‐S apocluster transporter) have essential functions outside of the metabolic network, perhaps for the supply of Fe‐S clusters for essential non‐enzyme proteins.

Despite their essentiality, the hemizygous mutants of these genes in the diploid BY4743 genetic background did not produce any significant growth defects (*p* < 0.01) with the specific growth rate for all mutants remaining within $0.43\pm 0.01\text{​}h^{-1}$ as also indicated by the model predictions. Our experiments demonstrated that the hemizygous mutants did not display significant differences (*p* < 0.01) with respect to their utilisation of glucose or ammonium as carbon and nitrogen sources, respectively, nor in their production of ethanol or glycerol. On average, the hemizygous mutants were observed to consume more iron per unit optical density equivalent number of cells although this difference was not observed to be significant due to the high variance between replicates. However, a significant difference in the intracellular distribution of copper was observed between these mutants and the wild type was observed. That said, measurements of intracellular haem, copper, reduced or total iron content of the mutants gave no further insight into the essential roles *of ARH1* , *YFH1* and *ATM1* inside or outside metabolism (Supporting Information S5).

The reactions with non‐zero fluxes were more often catalysed by enzymes encoded by essential genes (2.32‐fold over‐enrichment; *p* < 10^–40^) in Y7.Fe than in Y7.6. In line with this observation, *ARH1* was widely associated with reactions having non‐zero fluxes in the metabolic network. *ARH1* being an essential gene also for the model, the reorganisation of the fluxes in the network could only be investigated by introducing an “in silico reduction of function.” We used flux as a proxy for the functional capacity of the enzyme catalysing that reaction and lowered the flux value by 50% to mimic the impact of heterozygosity. The flux bounds that were set to 50% of their wild‐type flux value did not relate to nutrient uptake fluxes; therefore, they did not change the nutrient limitation of the system and this has been confirmed by the glucose uptake flux being maintained at its upper bound limit under both conditions. Consequently, the predicted growth rate was reduced by 50% in response to limiting the flux through the reaction catalysed by *ARH1* at 50% of its original value, the expected outcome for the remaining non‐zero‐fluxes was to be reduced by 50%, or to remain unchanged. However, 14% of all fluxes were observed to be rewired or had changed by a factor other than a 50% reduction.

Some 22% of the enzymes and transporters represented in Y7.Fe (963 in total) associated with 506 fluxes were affected by these changes. The rewiring of the fluxes was predominantly observed to involve lipid metabolism (*p* < 10^–11^). Furthermore, fluxes were observed to be rewired away from the metabolism of glycine and serine family of amino acids (*p* < 10^−6^) towards the metabolism of neutral lipids (*p* < 10^−4^). The enzymes associated with the reactions that displayed unexpected variations in the magnitude of their fluxes were significantly associated with oxidative phosphorylation (*p* < 10^–40^), aerobic respiration (*p* < 10^–30^), and purine nucleotide metabolism (*p* < 10^–72^).

Among the reactions with unexpectedly altered fluxes, 28% were orphan reactions, mostly involved in small molecule transport or exchange, for which the relevant gene has yet to be identified. Many of these reactions were involved with the transport or exchange of lipid metabolism intermediates across organelles, as well as of amino acids including glycine, l‐alanine, l‐leucine, serine and valine and of small inorganic molecules including ammonia, bicarbonate, carbon dioxide, hydronium ion, oxygen, phosphate and water (Supporting Information 3).

The rewiring of lipid metabolism and the changes in the fluxes in the energy pathways in response to a perturbation induced to mimic the reduction of *ARH1* function in the cell was in line with earlier findings on its role in biological systems. Apart from acting as an essential component of the ISC machinery, the Arh1 protein is an ortholog of the human adrenodoxin reductase (Manzella, Barros, & Nobrega, 1998), which was reported to function in the mitochondrial electron transfer chain that catalyses the conversion of cholesterol into pregnenolone. Expression of human *ARH1* on a retroviral vector was shown to restore the LDL receptor function in cells from patients suffering from familial hypercholesterolemia (Eden et al., 2002), demonstrating the enzyme’s role in neutral lipid metabolism, as also captured by our analysis in the model yeast system.

Because iron cofactors, specifically Fe‐S clusters, were used extensively as cofactors by enzymes catalysing mitochondrial reactions (particularly those that affect energy generation routes *via* aerobic respiration), constraining the flux of such a reaction was observed to lead to a reduction in the oxygen uptake flux, also by 50%. This was observed not only upon imposing limitations on iron uptake, but also upon rendering of the Fe‐S cluster biogenesis fluxes low, as demonstrated by the simulations carried out to mimic the impact of genetic modifications on the metabolic network. This example also demonstrated the tight links across the metabolic network. Because the flow of material (i.e. flux) through the reactions, which were catalysed by iron‐requiring enzymes, would not be isolated from the flux through those that did not require iron‐bearing enzymes. The fluxes through reactions which (in theory) had no direct relationship with iron metabolism were also affected in response to this extension of the model.

The extended model of the yeast metabolic network now serves as a functional platform to study rare disorders associated with the iron metabolism, as well as those of other ions to the extent of their involvement in iron‐associated pathways. *ARH1*, studied extensively above, encodes the yeast homolog of the adrenodoxin reductase, and mutations in the human gene are reported to be responsible for auditory neuropathy and optic atrophy (Paul et al., 2017). The model also allowed us to simulate the impairment of *CCC2*, the copper‐transporting ATPase, mutations in whose human homolog, *ATP7B*, is responsible for Wilson’s disease (Cankorur‐Cetinkaya et al., 2013; Júlvez, Dikicioglu, & Oliver, 2018). Using the model, we were able to demonstrate a critical impairment associated with this disorder—namely, that under conditions of high oxygen availability, the deletion of *CCC2* impaired growth in the absence of copper supplementation (Table 3).

## 4 DISCUSSION

The complex network of interactions between enzymes, metabolites and their regulators defines transitions between metabolic states. Metabolic state shifts mediate adaptive responses that allow cells to respond to environmental or genetic perturbations. Such shifts in the state of metabolism may be desirable in some cases, such as biotechnological applications, or most unwelcome in other situations such as switching to a diseased state that may induce cancer or other conditions. Genome‐scale models of the metabolic network are of central importance since they allow us to achieve a better understanding of how the fluxes can be rewired in response to an internal or external input, and thus have been frequently used in biotechnological applications as a prediction tool, since the aim is often to obtain a preferred flux distribution (Kerkhoven, Lahtvee, & Nielsen, 2014). Iron has an important role in a range of biotechnological applications from improving human nutrition (Puig, Andres‐Ccolas, Garcia‐Molina, & Penarrubia, 2007) to the production of recombinant proteins (Eck et al., 2018), and healthcare applications concerning iron storage abnormalities (Valerio, 2007). The incorporation of an extensive, well‐curated iron metabolism in metabolic networks may greatly enhance the opportunities metabolic models offer for applications in red, green, or white biotechnology.

This paper has presented a comprehensive extension of the genome‐scale stoichiometric model of the yeast metabolic network by incorporating the involvement of ion cofactors, which are not, themselves, consumed in the metabolic reactions in which they are involved. We selected the iron ion as our test case as it is involved in nearly 10% of all enzymatic steps in the yeast metabolic network. A substantial number of non‐metabolic proteins also recruit iron cofactors. Although iron species to be used by non‐metabolic proteins can be produced by the metabolic network model, they remain as dead‐end metabolites since these models do not include non‐metabolic biological processes. In its current form, introducing the empirical iron requirement of yeast as a constraint for the metabolic network would yield an inevitable overestimation of the iron requirement for metabolism, since all of the iron transported across the cell boundary can theoretically be utilised metabolically. Nevertheless, the quantitation of distinct iron‐containing cofactor species contributing to the biomass would allow an improved definition of biomass composition to be established, which would indirectly account for the iron species requirements of non‐metabolic proteins, resolving this problem of overestimation.

Although this study was specific to iron, we contend that our work has provided a new approach for handling co‐enzymes and co‐factors in stoichiometric models of metabolism, and that this approach can be generalised for other such entities. We made use of pseudo‐metabolites, which were not produced or consumed by the cell, in modelling the system. We used this formalism since these intermediates, functioning in cyclic interconversion pathways, allowed a more explicit description of the molecular mechanisms represented in the model. The Y7.Fe model allowed us to define the optimal turnover rate of iron cofactors in the metabolic network, something which is not possible to achieve experimentally using current analytical technologies. The method provided a means to estimate enzyme fluxes from reaction fluxes and cofactor availability. Proteomics data available at the global scale can, at present, only provide us with the relative quantitation of some enzymes; therefore, empirical protein abundance data do not afford us the means to evaluate our predictions. However, we note once again that ion cofactor availability, rather than empirically determined enzyme turnover rates, was more prominent as a limiting factor in determining the predictions that can be made using the Y7.Fe model. Therefore, we believe our method provides a very reasonable estimate of the iron‐recruiting enzyme requirements of the metabolic network under defined circumstances, which is currently not possible with any other method.

Our model represents an unconventional way of extending metabolic network models to incorporate non‐metabolic information. The ion cofactors are “non‐metabolic” in the sense that they are conserved moieties that are not, themselves, transformed by metabolic reactions. Although they can be considered as “pseudometabolites,” these cofactors proved highly effective in altering the distribution of fluxes through the metabolic network. The flux distribution would not have been substantially affected were it not for the involvement of cofactors taken up and biosynthesized by the new reactions introduced into the metabolic network model. This distribution can now be constrained not only by the macronutrient (e.g. glucose) availability, but also by the availability of an essential micronutrient, that is iron. The ability to incorporate the roles of such ion cofactors could prove essential for constructing models of biological systems at the level of the whole cell or organism.

## CONFLICTS OF INTEREST

The authors declare that there are no conflicts of interest.

## Supporting information

Supplementary information

Supplementary information

Supplementary information

Supplementary information

Supplementary information

Supplementary information

Supplementary information

Supplementary information

Supplementary information

Supplementary information

Supplementary information

Supplementary information
