# Supplementary material for: Extension of the yeast metabolic model to include iron metabolism and its use to estimate global levels of iron‐recruiting enzyme abundance from cofactor requirements
Source: Biotechnol Bioeng. 2019 Jan 12;116(3):610–21. doi: 10.1002/bit.26905 (PMC6492170; doi:10.1002/bit.26905)
Supplement: Supplementary file 3 — Supplementary information [file BIT-116-610-s003.docx]

**Supplemental Methods**

**1 Modelling Methods**

**1.1 Model extension annotations and simulation environment**

The extension annotations were documented in compliance with the MIRIAM standards (Le Novère et al., 2005); ChEBI (Hastings et al., 2013) and KEGG (Kanehisa et al., 2014) identifiers (IDs) of the compounds, KEGG (Kanehisa et al., 2014) and SGD (Cherry et al., 2012) IDs of the genes, UniProt (The UniProt Consortium, 2013) IDs of the enzymes, and KEGG (Kanehisa et al., 2014) IDs of the reactions were documented whenever applicable. PUBMED IDs (Roberts, 2001) of the relevant publications were provided as the resource if KEGG reaction IDs were not available. Logic rules for multiple genes encoding enzymes involved in the catalysis of a single reaction were derived from the literature (see Supporting Information S2 in supplemental material). Any signal transduction event was designated by the SBO:0000464 term: “state variable” in the model.

Model simulations were carried out in the MATLAB environment R2016b (9.1.0.441655, Mathworks, USA) with the COBRA Toolbox (v3.1.2), the SBML Toolbox v4.1.0 and libSBML library v5.15.0 running in the background employing standard linear optimisation techniques (Gurobi 8.0.1 - http://www.gurobi.com/). The COBRA Toolbox 2.0 Protocol Exchange

(<https://www.nature.com/protocolexchange/protocols/2097#/procedure>)

is provided as a guide to the different commands employed in this study (Hyduke et al., 2011). The objective function was to maximise growth.

It should be noted that the COBRA toolbox does not print the value for the same metabolite employed as reactant and product. However, this does not mean that it is not saved by COBRA, based on the observations made on the model simulations. The iron species represented as cofactors in the reactions are only listed among the products in their respective reactions. Nevertheless, there is flux through these reactions, and were these species only “produced” as implicated by COBRA reactant and product lists, since the model does not have an iron export flux, this situation should have had led to iron accumulation in the cell, and consequently rendered t the system infeasible. This was shown not to be the case, therefore the iron within the cell must be recycled to compensate for this. So, although iron species do not appear as reactants, this does not mean that they are not considered as reactants in the metabolic network. To provide further support for this notion, we have conducted an analysis where the same cofactor was identified with different species IDs, which were interconvertible. By doing so, the COBRA Toolbox indeed does recognise the same species with different IDs; however, the internal recycling of one species into the other modifies the system for each iron species to form a cyclic conversion, through which no flux needs to flow, in line with what is observed in other cyclic conversions in the model, for example, in flavin nucleotide interconversions. ***The system does not need iron uptake, and renders that flux zero, taking the model back to its original form (Y7.6), in practice.

The performance of Gurobi when working with close-to-zero values was evaluated in two ways: Since the utilization of values less than 10^-10^ would not be preferable and was thought to be treated as 0 in many applications, we tested this notion by rendering the iron uptake flux zero since this treatment of low numbers as zero would indicate that none of the metabolic reactions would require iron and, consequently, changing the iron uptake flux should make no difference to the simulations. However, in line with empirical data, iron was essential for the yeast 7.Fe metabolic network model; simulations in which iron uptake was not allowed did not yield any growth, indicating the non-zero treatment of the low stoichiometric coefficients by Gurobi. Additionally, we carried out performance tests on Gurobi to evaluate/highlight any potential issues that the close-to-zero numbers in the model would introduce. No numerical issues were identified in logs, and the condition number was sufficiently low as to suggest that there were no numerical issues with the use of close-to-zero values in the model. We further evaluated the performance by varying the “NumericFocus” parameter. Increasing the setting from 1 up to 3, despite extending the analysis time substantially, did not introduce changes affecting the LP solution at an integer placed at larger than the 5th significant digit.

**1.2 Simulation approaches**

In addition, a performance test was carried out to observe the effects of bounding constraints whenever available. Glucose uptake was assumed as 1mmol$\cdot$(g biomass hr)^-1^ unless otherwise specified. Copper and iron requirements were adopted from (Baganz, Hayes, Marren, Gardner, & Oliver, 1997). Only the maximum upper limits of the uptake reactions were constrained as indicated by the medium compositions. Iron or copper were considered as “low” at 10% of their concentrations in the original medium formulation, and the system was thus constrained to maximally uptake the specified amount from the extracellular environment; r_1387, r_1861, and r_1388 constrained for the uptake of ironIII, ironII, and copperII, respectively. This action does not attempt to simulate situations where the metabolic network becomes limited for iron or copper; rather, it aims to investigate how high- and low-affinity uptake systems in the metabolic network would behave *in silico* in response to high (i.e. above the threshold that would activate high-affinity ion uptake) or low (i.e. below the threshold that would activate high-affinity ion uptake) extracellular availability of iron or copper. The flux bounds (v) were derived from metabolite concentrations, [c] (g/L) by

$v \left( mmol {g biomass}^{-1}h^{-1} \right)=\frac{\left[ c \right]\left( gL^{-1} \right)}{molecular weight \left( g{mol}^{-1} \right)}\times\frac{1000 mmol}{1 mol}\times\frac{1}{biomass \left( gL^{-1} \right)}\times growth rate\left( h^{-1} \right).$

The simulations assumed a constant growth at a rate of 0.1 hr^-1^. Low-affinity uptake routes were blocked when simulating low extracellular iron availability. The flux through a reaction was constrained by setting both the upper and the lower bounds of a reaction to a given/selected value. For representing heterozygosity, a 50% reduction in the functionality of an enzyme was assumed to decrease the flux through the reactions it catalyses by 50%. Therefore, the fluxes through those reactions were constrained at 50% of the value under unperturbed simulation conditions. For those experiments where heterozygosity was investigated, experimental measurements on metabolites were also employed to constrain the fluxes. Ethanol and glycerol production fluxes per unit cell mass, which were calculated from the metabolite concentrations and growth rate, were employed to constrain the upper bound of the ethanol and glycerol export fluxes, using a similar approach to that adopted by (Dikicioglu, Kırdar, & Oliver, 2015).

Flux variabilities were considered in comparing the flux distributions obtained by Y7.Fe and Y7.6 as follows: For each model, the minimum and the maximum value that each individual flux could take was determined by flux variability analysis and any flux was considered different between the predictions obtained by two models if, and only if, the magnitude of that difference was greater than the magnitude of the difference between the minimum and the maximum value that flux could potentially take in either of the two models.

**1.3 Cofactor representation**

Iron ions, copper ions, pyridoxine, haem entities and Fe-S clusters were integrated as cofactors (*X*) in the model. The stoichiometry of the cofactors required for each reaction was designated by x. Although technically displayed as substrates and products, this representation prevents the actual production or depletion of any enzyme cofactors, through the maintenance of constant stoichiometric coefficients. This implementation allowed correct use of the relevant cofactors (X) of enzyme (N) without being produced or consumed in any reaction. The number of active sites on N was assumed to be 1 in all cases due to the unavailability of data. It may be that this leads to the overestimation of fluxes through specific reactions.

**1.4 Evaluation of the predictive power of the model**

A Boolean criterion was adopted for the presence or absence of growth, where a positive or a negative call was defined as the presence (= 1) or absence (= 0) of growth, respectively. A true positive (TP) was a non-essential gene deletion mutant predicted as viable and a true negative (TN) was an essential gene deletion mutant predicted as inviable. A false positive (FP) designated a viable prediction for the deletion of an essential gene, whereas a false negative (FN) was assigned when the model yielded an inviable prediction for the deletion of a non-essential gene. The following success measures were employed to assess the predictions: $positive predictive value \left( PPN \right)= \frac{TP}{TP + FP}$ , $negative predictive value \left( NPN \right)= \frac{TN}{TN + FN}$, $sensitivity= \frac{TP}{TP + FN}$ , $specificity= \frac{TN}{TN + FP}$, and $predictive success= \frac{TP + TN}{TP + TN + FP+ FN}$. Hypergeometric p-values were determined based on the cumulative distribution functions for determining under- or over-enrichment of factors.

**2 Experimental Methods**

**2.1 Cultivation conditions, the subcellular fraction enrichment protocols, and analytical assays**

Three separate cultures were grown to an OD_600_ of 0.7 at 30°C in YPD medium, allowing sufficient aeration in vented-cap tissue culture flasks with low protein binding (TPP®; surface area (cm^2^) to height ratio (cm) = 300:4.5) with shaking (220 rpm). Fresh medium from the same batch was employed in further analytical assays. Culture supernatants were collected by centrifugation at 7.6k rpm for 10 min at 4^o^C. Cell wall digestion and lysis of the harvested cells (1 OD_600_ equivalent) was carried out as described in Qiagen DNeasy Blood & Tissue Kit protocol, and crude cell extracts as well as the supernatant was stored at -20^o^C. Yeast Mitochondria Isolation Kit from Sigma-Aldrich (Cat no: MITOISO3) was used for the isolation of an enriched mitochondrial fraction from yeast cells starting from 20 OD_600_ equivalent culture and intact mitochondria were stored -20^o^C until further use. The cytosol was separated from the cell’s organelles by passage through an oil layer, employing AbCam’s Cytosol/Particulate Rapid Separation Kit (Cat no: ab65398) from a 0.6 OD_600_ equivalent culture. A vacuole-enriched fraction was isolated from 420 OD_600_ equivalent yeast cells following a yeast-specific protocol as described (Rieder & Emr, 2001). Proteins in all fractions were precipitated in 20%w/v trichloroacetic acid at 4^o^C (Bolstad, Botelho, & Wood, 2010), and protein free-lysates were analysed separately.

The glucose, glycerol, ethanol and ammonium content of the supernatant were determined enzymatically by r-biopharm Roche Yellow Line assays (Cat nos: 10716251035, 10148270035, 10176290035, and 11112732035, respectively). Copper content, the total haem content, and the Fe^2+^ and total iron content of the samples post -reduction were determined colorimetrically employing the Copper Assay Kit from Sigma-Aldrich (Cat no: MAK127), BioAssay Systems QuantiChrom^TM^ Heme Assay Kit (Cat no: DIHM-250), and the Iron Assay Kit by Sigma-Aldrich (Cat no: MAK025), respectively, as described by their manufacturers. Assays were executed in 96-well Corning® Costar® 96-well flat–bottom cell culture plates whenever applicable.

***References***

Baganz, F., Hayes, A., Marren, D., Gardner, D. C., & Oliver, S. G. (1997). Suitability of replacement markers for functional analysis studies in Saccharomyces cerevisiae. *Yeast (Chichester, England)*, *13*(16), 1563–73. http://doi.org/10.1002/(SICI)1097-0061(199712)13:16<1563::AID-YEA240>3.0.CO;2-6

Bolstad, H. M., Botelho, D. J., & Wood, M. J. (2010). Proteomic analysis of protein-protein interactions within the cysteine sulfinate desulfinase Fe-S cluster biogenesis system. *Journal of Proteome Research*, *9*(10), 5358–69. http://doi.org/10.1021/pr1006087

Cherry, J. M., Hong, E. L., Amundsen, C., Balakrishnan, R., Binkley, G., Chan, E. T., … Wong, E. D. (2012). Saccharomyces Genome Database: the genomics resource of budding yeast. *Nucleic Acids Research*, *40*(Database issue), D700-5. http://doi.org/10.1093/nar/gkr1029

Dikicioglu, D., Kırdar, B., & Oliver, S. G. (2015). Biomass composition: the “elephant in the room” of metabolic modelling. *Metabolomics*, *11*(6). http://doi.org/10.1007/s11306-015-0819-2

Hastings, J., de Matos, P., Dekker, A., Ennis, M., Harsha, B., Kale, N., … Steinbeck, C. (2013). The ChEBI reference database and ontology for biologically relevant chemistry: enhancements for 2013. *Nucleic Acids Research*, *41*(Database issue), D456-63. http://doi.org/10.1093/nar/gks1146

Hyduke, D., Hyduke, D., Schellenberger, J., Que, R., Fleming, R., Thiele, I., … Palsson, B. (2011). COBRA Toolbox 2.0. *Protocol Exchange*. http://doi.org/10.1038/protex.2011.234

Kanehisa, M., Goto, S., Sato, Y., Kawashima, M., Furumichi, M., & Tanabe, M. (2014). Data, information, knowledge and principle: back to metabolism in KEGG. *Nucleic Acids Research*, *42*(Database issue), D199-205. http://doi.org/10.1093/nar/gkt1076

Le Novère, N., Finney, A., Hucka, M., Bhalla, U. S., Campagne, F., Collado-Vides, J., … Wanner, B. L. (2005). Minimum information requested in the annotation of biochemical models (MIRIAM). *Nature Biotechnology*, *23*(12), 1509–15. http://doi.org/10.1038/nbt1156

Rieder, S. E., & Emr, S. D. (2001). Isolation of subcellular fractions from the yeast Saccharomyces cerevisiae. *Current Protocols in Cell Biology*, *8*, 1–68.

Roberts, R. J. (2001). PubMed Central: The GenBank of the published literature. *Proceedings of the National Academy of Sciences of the United States of America*, *98*(2), 381–2. http://doi.org/10.1073/pnas.98.2.381

The UniProt Consortium. (2013). Update on activities at the Universal Protein Resource (UniProt) in 2013. *Nucleic Acids Research*, *41*(Database issue), D43-7. http://doi.org/10.1093/nar/gks1068
