# Supplementary material for: Extension of the yeast metabolic model to include iron metabolism and its use to estimate global levels of iron‐recruiting enzyme abundance from cofactor requirements
Source: Biotechnol Bioeng. 2019 Jan 12;116(3):610–21. doi: 10.1002/bit.26905 (PMC6492170; doi:10.1002/bit.26905)
Supplement: Supplementary file 6 — Supplementary information [file BIT-116-610-s006.docx]

| **ORF** | **Gene** | **Compartment** | **# of Fe-S clusters** | **Type of Fe-S cluster** | **Reference** |
| --- | --- | --- | --- | --- | --- |
| YNL240c | *NAR1* | Cytosol | 2 | 4Fe-4S, 4Fe-4S | (1) |
| YGL091c | *NBP35* | Cytosol | 2 | 4Fe-4S, 4Fe-4S | (2) |
| YGL009c | *LEU1* | Cytosol | 1 | 4Fe-4S | (3) |
| YJR137c | *ECM17^a^* | Cytosol | 1 | 4Fe-4S | (3) |
| YPL086c | *ELP3^a^* | Cytosol | 1 | 4Fe-4S | (3) |
| YPL207w | *TYW1^a^* | Cytosol | 1 | 4Fe-4S | (4) |
| YDR091c | *RLI1^a^* | Cytosol | 2 | 4Fe-4S, 4Fe-4S | (3) |
| YDR091c | *RLI1^a^* | Nucleus | 2 | 4Fe-4S, 4Fe-4S | (3) |
| YER171w | *RAD3^a^* | Nucleus | 1 | 4Fe-4S | (5) |
| YOL043c | *NTG2^a^* | Nucleus | 1 | 4Fe-4S | (3) |
| YKL045w | *PRI2* | Nucleus | 1 | 4Fe-4S | (6) |
| YLL041c | *SDH2* | Mitochondrion | 3 | 2Fe-2S, 4Fe-4S,3Fe-4S | (3) |
| YEL024w | *RIP1* | Mitochondrion | 1 | 2Fe-2S | (3) |
| YLR304c | *ACO1* | Mitochondrion | 1 | 4Fe-4S | (3) |
| YDR234w | *LYS4* | Mitochondrion | 1 | 4Fe-4S | (3) |
| YJR016c | *ILV3* | Mitochondrion | 1 | 4Fe-4S | (3) |
| YPL252c | *YAH1* | Mitochondrion | 1 | 2Fe-2S | (3) |
| YOR196c | *LIP5^a^* | Mitochondrion | 2 | 4Fe-4S, 4Fe-4S | (3) |
| YGR286c | *BIO2* | Mitochondrion | 2 | 2Fe-2S, 4Fe-4S | (3) |
| YDL171c | *GLT1* | Mitochondrion | 1 | 4Fe-4S | (3) |

**Table S6.** Fe-S cluster containing proteins in *Saccharomyces cerevisiae*

^a^ Not employed in Yeast7.6.

***References***

1. Urzica E, Pierik AJ, Mühlenhoff U, Lill R. 2009. Crucial Role of Conserved Cysteine Residues in the Assembly of Two Iron−Sulfur Clusters on the CIA Protein Nar1. Biochemistry 48:4946–4958.

2. Netz DJA, Pierik AJ, Stümpfig M, Mühlenhoff U, Lill R. 2007. The Cfd1–Nbp35 complex acts as a scaffold for iron-sulfur protein assembly in the yeast cytosol. Nat Chem Biol 3:278–286.

3. Lill R, Dutkiewicz R, Elsässer H-P, Hausmann A, Netz DJA, Pierik AJ, Stehling O, Urzica E, Mühlenhoff U. 2006. Mechanisms of iron–sulfur protein maturation in mitochondria, cytosol and nucleus of eukaryotes. Biochim Biophys Acta - Mol Cell Res 1763:652–667.

4. Noma A, Kirino Y, Ikeuchi Y, Suzuki T. 2006. Biosynthesis of wybutosine, a hyper-modified nucleoside in eukaryotic phenylalanine tRNA. EMBO J 25:2142–2154.

5. Rudolf J, Makrantoni V, Ingledew WJ, Stark MJR, White MF. 2006. The DNA Repair Helicases XPD and FancJ Have Essential Iron-Sulfur Domains. Mol Cell 23:801–808.

6. Klinge S, Hirst J, Maman JD, Krude T, Pellegrini L. 2007. An iron-sulfur domain of the eukaryotic primase is essential for RNA primer synthesis. Nat Struct Mol Biol 14:875–877.
