# Supplementary material for: Extension of the yeast metabolic model to include iron metabolism and its use to estimate global levels of iron‐recruiting enzyme abundance from cofactor requirements
Source: Biotechnol Bioeng. 2019 Jan 12;116(3):610–21. doi: 10.1002/bit.26905 (PMC6492170; doi:10.1002/bit.26905)
Supplement: Supplementary file 7 — Supplementary information [file BIT-116-610-s007.docx]

**Table S7**. Iron family cofactors in the existing metabolic network model (Y7.6) of yeast

| Activity | Reaction ID in Y7.6 | M / A^a^ | Enzyme/Complex | Cofactor | Cofactors enzyme^-1^ |
| --- | --- | --- | --- | --- | --- |
| C-4 methyl sterol oxidase | r_0238 r_0239 r_0240 r_0241 | M | Erg25p | iron III^b^ | 2 |
| C-5 sterol desaturase | r_0242 | M | Erg3p | iron III^b^ | 2 |
| ribonucleotide reductase | r_0974 r_0975 r_0976 r_0977 r_0978 r_0979 | M | Rnr1p Rnr2p  Rnr3p Rnr4p | iron III^b^ | 2 |
| sphinganine C4-hydroxylase | r_0259 r_0261 r_0922 | M | Sur2p^c^ | iron III  haem a | 1  1 |
| sphingolipid alpha-hydroxylase | r_0260 r_0262 r_0267  r_0268 r_0269 r_0270 | M | Scs7p^c^ | iron III  haem a | 1  1 |
| delta(9) fatty acid desaturase | r_2182 r_2183 | M | Ole1p^c^ | iron III  haem a | 1  1 |
| multi-copper oxidase | r_1288 | A | Fet3p | copper I | 4 |
| multi-copper oxidase | r_1305 | A | Fet5p | copper I | 4 |
| ferrocytochrome-c: O-oxidoreductase | r_0439 | M | Mitochondrial Respiratory Chain Complex III | copper I  haem c  ferrohaem b | 1  2  2 |
| cytochrome c, isoforms 1 and 2 | r_0001 r_0002 r_0004  r_0437 | M | Cyc1p Cyc7p | haem a | 1 |
| C-22 sterol desaturase | r_0233 | M | Erg5p | haem a | 1 |
| cytosolic catalase T | r_0255 | M | Ctt1p | haem a | 1 |
| catalase A | r_0256 | M | Cta1p | haem a | 1 |
| lanosterol 14-alpha-demethylase | r_0317 | M | Erg11p | haem a | 1 |
| ferrocytochrome-c: O-oxidoreductase | r_0438 | M | Mitochondrial Respiratory Chain Complex IV | haem a | 2 |
| L-tryptophan: oxygen 2,3-oxidoreductase | r_0694 | M | Bna2p | haem a | 1 |
| N-formyltyrosine oxidase | r_0763 r_0920 | M | Dit2p | haem a | 1 |
| ferric and cupric reductases | r_1284 | A | Fre1p Fre2p | haem a | 1 |
| ferric reductase | r_1296 | A | Fre6p | haem a | 1 |
| ferric reductase | r_1302 | A | Fre5p | haem a | 1 |
| ferric reductase | r_1334 r_1337 r_1340 r_1343 r_1346 | A | Fre1p Fre2p  Fre3p Fre4p | haem a | 1 |
| sulphite reductase | r_1027 | M | Met5p Met10p | sirohaem | 1 |
| cytochrome b2 | r_0004 | M | Cyb2p | ferrohaem b | 1 |
| cytochrome c peroxidase | r_0437 | M | Ccp1p | ferrohaem b | 1 |
| succinate dehydrogenase | r_1021 | M | Mitochondrial Respiratory Chain Complex II | ferrohaem b | 1 |
| 5-aminolevulinate synthase | r_0081 | M | Hem1p | pyridoxine | 1 |
| 2-isopropylmalate hydratase | r_0023 r_0060 | M | Leu1p | 4Fe-4S | 1 |
| 2-methylcitrate dehydratase | r_0027 r_0542 | M | Lys4p | 4Fe-4S | 1 |
| aconitate hydratase | r_0280 r_0302 r_2305 | M | Aco1p | 4Fe-4S | 1 |
| dihydroxy-acid dehydratase | r_0352 | M | Ilv3p | 4Fe-4S | 1 |
| glutamate synthase | r_0472 r_0761 | M | Glt1p | 4Fe-4S | 1 |
| sulphite reductase | r_1027 | M | Met5p | 4Fe-4S | 1 |
| CIA related Fe/S maturation | r_1374 r_1375 | A | Nbp35p | 4Fe-4S 4Fe-4S | 2 |
| CIA related Fe/S maturation | r_1376 r_1377 | A | Nar1p | 4Fe-4S 4Fe-4S | 2 |
| succinate dehydrogenase | r_1021 | M | Sdh2p (Mitochondrial Respiratory Chain Complex II) | 2Fe-2S 4Fe-4S  3Fe-4S | 3 |
| biotin synthase | r_0229 | M | Bio2p | 2Fe-2S 4Fe-4S | 2 |
| ferrocytochrome-c: O-oxidoreductase | r_0439 | M | Rip1p (Mitochondrial Respiratory Chain Complex III) | 2Fe-2S | 1 |
| ISC machinery for Fe/S biogenesis, haem O monooxygenase | r_0530 r_1349 r_1350 | M + A | Yah1p | 2Fe-2S | 1 |

^a^ M: modified, A: added

^b^ Erg3p and Erg25p were both reported to incorporate oxo-diiron (Fe-O-Fe) species (1, 2), so were the ribonucleotide reductases Rnr1-4p (3). Oxo-diiron species were reported to carry FeIII charge (4).

^c^ These enzymes are accepted as oxo-diiron enzymes of the fatty acid hydroxylase/sterol desaturase family, and are also referred to as a specific subgroup as heme/diiron enzymes (2).

**References**

1. Li L, Kaplan J. 1996. Characterization of yeast methyl sterol oxidase (ERG25) and identification of a human homologue. J Biol Chem 271:16927–33.

2. Shakoury-Elizeh M, Protchenko O, Berger A, Cox J, Gable K, Dunn TM, Prinz WA, Bard M, Philpott CC. 2010. Metabolic response to iron deficiency in Saccharomyces cerevisiae. J Biol Chem 285:14823–33.

3. Hohenberger J, Ray K, Meyer K. 2012. The biology and chemistry of high-valent iron–oxo and iron–nitrido complexes. Nat Commun 3:720.

4. Kurtz DM. 1990. Oxo- and hydroxo-bridged diiron complexes: a chemical perspective on a biological unit. Chem Rev 90:585–606.
