# Supplementary material for: Extension of the yeast metabolic model to include iron metabolism and its use to estimate global levels of iron‐recruiting enzyme abundance from cofactor requirements
Source: Biotechnol Bioeng. 2019 Jan 12;116(3):610–21. doi: 10.1002/bit.26905 (PMC6492170; doi:10.1002/bit.26905)
Supplement: Supplementary file 8 — Supplementary information [file BIT-116-610-s008.docx]

**Table S8** Excluded protein - iron family cofactor associations

| **Activity** | **Enzyme** | **Reason for exclusion** | **Cofactor** | **Cofactors enzyme^-1^** |
| --- | --- | --- | --- | --- |
| glutaredoxin 5 | Grx5p | does not constitute holo cluster | 2Fe-2S | 1 |
| scaffold protein | Isu1p | does not constitute holo cluster | 2Fe-2S | 1 |
| scaffold protein | Isu2p | does not constitute holo cluster | 2Fe-2S | 1 |
| bifunctional dehydrogenase and ferrochelatase | Met8p | conflicting data: involved in cytosolic sirohaem biosynthesis but reported to employ mitochondrial 2Fe-2S cluster | 2Fe-2S | 1 |
| aconitase | Aco2p | absent from Y7.6 | 4Fe-4S | 1 |
| elongator complex protein 3 | Elp3p | non-metabolic, absent from Y7.6 | 4Fe-4S | 1 |
| scaffold protein | Nfu1p | does not constitute holo cluster | 4Fe-4S | 1 |
| DNA glycosylase | Ntg2p | non-metabolic, absent from Y7.6 | 4Fe-4S | 1 |
| DNA replication | Pri2p | non-metabolic, absent from Y7.6 | 4Fe-4S | 1 |
| DNA helicase | Rad3p | non-metabolic, absent from Y7.6 | 4Fe-4S | 1 |
| tRNA wybutosine-synthesising protein 1 | Tyw1p | non-metabolic, absent from Y7.6 | 4Fe-4S | 1 |
| lipoate synthase | Lip5p | absent from Y7.6 | 4Fe-4S  4Fe-4S | 2 |
| Rnase L inhibitor protein 1 | Rli1p | non-metabolic, absent from Y7.6 | 4Fe-4S  4Fe-4S | 2 |
| cytochrome b5 | Cyb5p | absent from Y7.6 | haem a | 1 |
| cytochrome c haem lyase | Cyc3p | absent from Y7.6 | haem a | 1 |
| cytochrome c1 haem lyase | Cyt2p | absent from Y7.6 | haem a | 1 |
| transcription factor with haem-dependent DNA-binding activity | Hap1p | regulatory component, absent from Y7.6 | haem a | 1 |
| putative protein of unknown function | Irc21p | only documented from metal binding motif similarity | haem a | 1 |
| copper chaperone for Sod1p | Ccs1p | copper metabolism absent from Y7.6 | copper I | 1 |
| cytochrome c oxidase subunit I | Cox1p | COX metallation not included in Y7.6 | copper I | 1 |
| cytochrome c oxidase subunit II | Cox2p | COX metallation not included in Y7.6 | copper I | 1 |
| copper chaperone | Cox17p | COX metallation not included in Y7.6 | copper I | 1 |
| metallothionein | Cup1-1p | copper metabolism absent from Y7.6 | copper I | 1 |
| metallothionein | Cup1-2p | copper metabolism absent from Y7.6 | copper I | 1 |
| copper binding transcription factor | Cup2p | regulatory component, absent from Y7.6 | copper I | 1 |
| Cup2p homolog transcriptional activator | Haa1p | regulatory component, absent from Y7.6 | copper I | 1 |
| copper sensing transcription factor | Mac1p | regulatory component, absent from Y7.6 | copper I | 1 |
| copper-transporting ATPase | Pca1p | copper metabolism absent from Y7.6 | copper I | 1 |
| copper-zinc superoxide dismutase | Sod1p | copper metabolism absent from Y7.6 | copper I | 1 |
| protein serine/threonine phosphatase | Glc7p | regulatory component, absent from Y7.6, UniProt only Mg/Mn evidence | Mg/Mn | 2 |
| protein serine/threonine phosphatase | Pph21p | regulatory component, absent from Y7.6, UniProt only Mg/Mn evidence | Mg/Mn | 2 |
| protein serine/threonine phosphatase | Pph22p | regulatory component, absent from Y7.6, UniProt only Mg/Mn evidence | Mg/Mn | 2 |
| protein serine/threonine phosphatase | Pph3p | regulatory component, absent from Y7.6, UniProt only Mg/Mn evidence | Mg/Mn | 2 |
| protein serine/threonine phosphatase | Ppt1p | regulatory component, absent from Y7.6, UniProt only Mg/Mn evidence | Mg/Mn | 2 |
| protein serine/threonine phosphatase | Ppz1p | regulatory component, absent from Y7.6, UniProt only Mg/Mn evidence | Mg/Mn | 2 |
| protein serine/threonine phosphatase | Ppz2p | regulatory component, absent from Y7.6, UniProt only Mg/Mn evidence | Mg/Mn | 2 |
| protein serine/threonine phosphatase | Sit4p | regulatory component, absent from Y7.6, UniProt only Mg/Mn evidence | Mg/Mn | 2 |
| protein serine/threonine phosphatase | Ppq1p | regulatory component, absent from Y7.6, UniProt only Mg/Mn evidence | Mg/Mn | 2 |
| protein serine/threonine phosphatase | Ppg1p | regulatory component, absent from Y7.6, UniProt only Mg/Mn evidence | Mg/Mn | 2 |
| nitric oxide oxidoreductase | Yhb1p | absent from Y7.6 | ferrohaem b | 1 |
