# Supplementary material for: Extension of the yeast metabolic model to include iron metabolism and its use to estimate global levels of iron‐recruiting enzyme abundance from cofactor requirements
Source: Biotechnol Bioeng. 2019 Jan 12;116(3):610–21. doi: 10.1002/bit.26905 (PMC6492170; doi:10.1002/bit.26905)
Supplement: Supplementary file 12 — Supplementary information [file BIT-116-610-s012.docx]

SUPPORTING INFORMATION CAPTIONS

**Supporting Information S1** Extended model of the metabolic network of yeast (SBML level 2, version 4) (file format: .xml).

**Supporting Information S2** Details on the extended model. The modifications carried out on the primary model, the new species types, and the new species (including both genes and metabolites) are provided in separate worksheets. The reactions that are introduced into the network are described and provided along with their gene associations accompanied by the logical rules for these genes (whenever applicable), the reversibility rules, the literature/database evidence for the reaction, and the upper and lower bounds of each reaction in the last worksheet (file format: .xlsx).

**Supporting Information S3** Text providing the details on the methods employed in modelling and experimental variation (file format: .docx).

**Supporting Information S4** Simulation code for the analyses conducted as described in the text (file format: .m).

**Supporting Information S5** Worksheet on flux distributions and predictions of viability. The evaluation of the distribution of fluxes between the Y7.Fe and Y7.6, the growth predictions for single-gene deletants and double-gene deletants, the distribution of fluxes for reduced flux through the reactions catalysed by Arh1p, and the flux variability analyses for determining the enzyme abundance upper- and lower-bounds are provided in separate tabs (file format: .xlsx).

**Supporting Information S6** Table summarising the Fe-S cluster requirements of metabolic enzymes employed in the metabolic network model Y7.6 (file format: .docx).

**Supporting Information S7** Table summarising the iron family cofactor requirements of the enzymes and active transporters of the Y7.6 yeast metabolic network model (file format: .docx).

**Supporting Information S8** Table summarising the enzyme-cofactor relationships excluded from this reconstruction and detailed reasons for exclusion (file format: .docx).

**Supporting Information S9** Detailed comparison of iron-ion binding enzymes in Y7.Fe and those reported in UniProt and those associated with the iron-ion binding Molecular Function Gene Ontology Term (GO:0005506) are provided in a single worksheet (file format: .xlsx).

**Supporting Information S10** Details on calculation of the new biomass equation with iron entities. Details regarding the calculation of the stoichiometric coefficients of iron entities in the biomass equation, and the flux distributions with the new model (denoted as Y7.FeBM) are provided in separate worksheets (file format: .xlsx).

**Supporting Information S11** Extended model of the metabolic network of yeast with iron entities incorporated into the biomass equation (SBML level 2, version 4) (file format: .xml).
